# Supplementary material for: Rat liver regeneration following ablation with irreversible electroporation
Source: PeerJ. 2016 Jan 21;4:e1571. doi: 10.7717/peerj.1571 (PMC4727979; doi:10.7717/peerj.1571)
Supplement: Supplemental Information 1 [file peerj-04-1571-s001.pptx]

## Slide 1
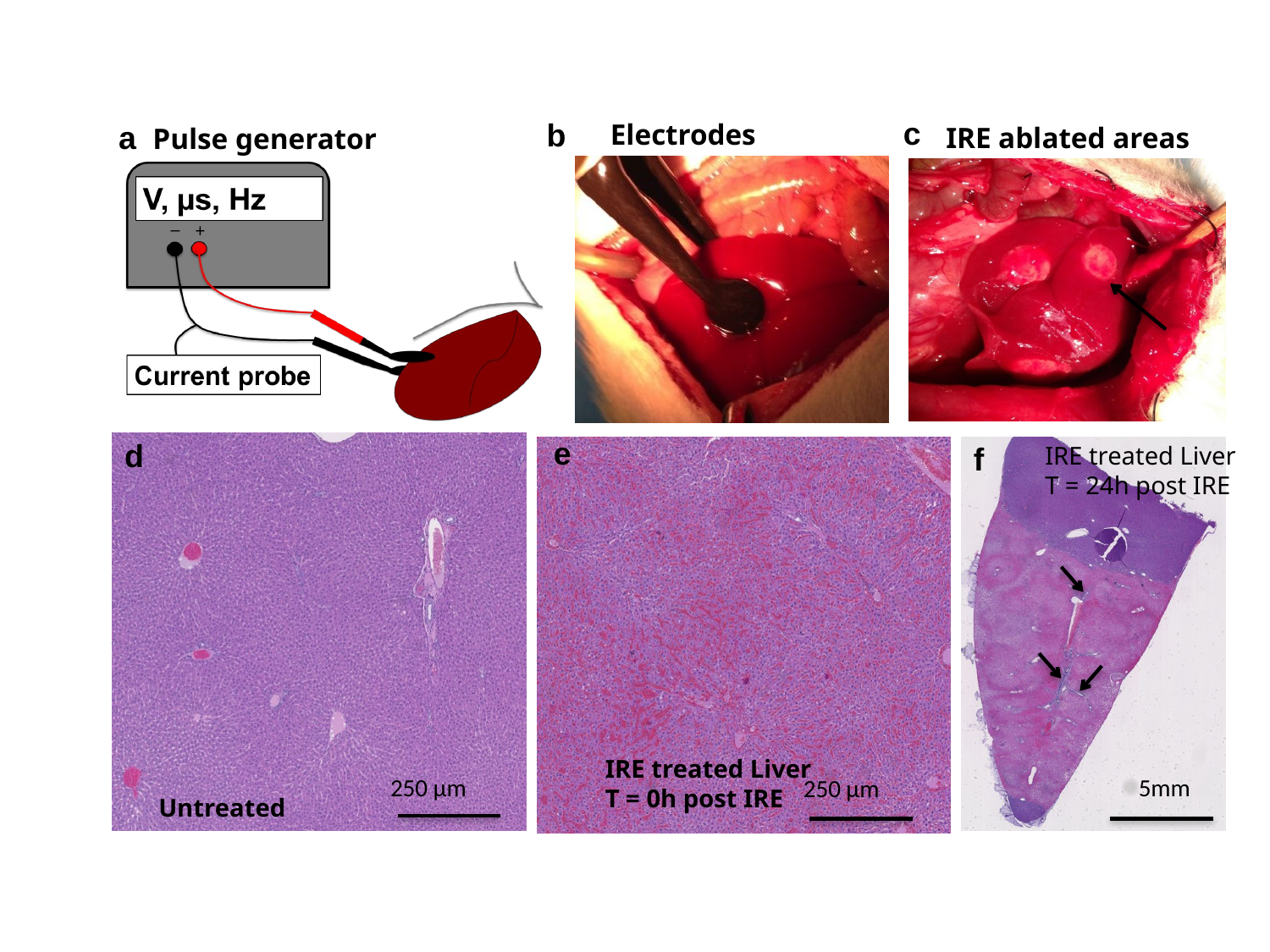

c
b
Electrodes
a
IRE ablated areas
Pulse generator
250 µm
IRE treated Liver
T = 24h post IRE
IRE treated Liver
T = 0h post IRE
250 µm
Normal Liver
5mm
Untreated
e
d
f

## Slide 2
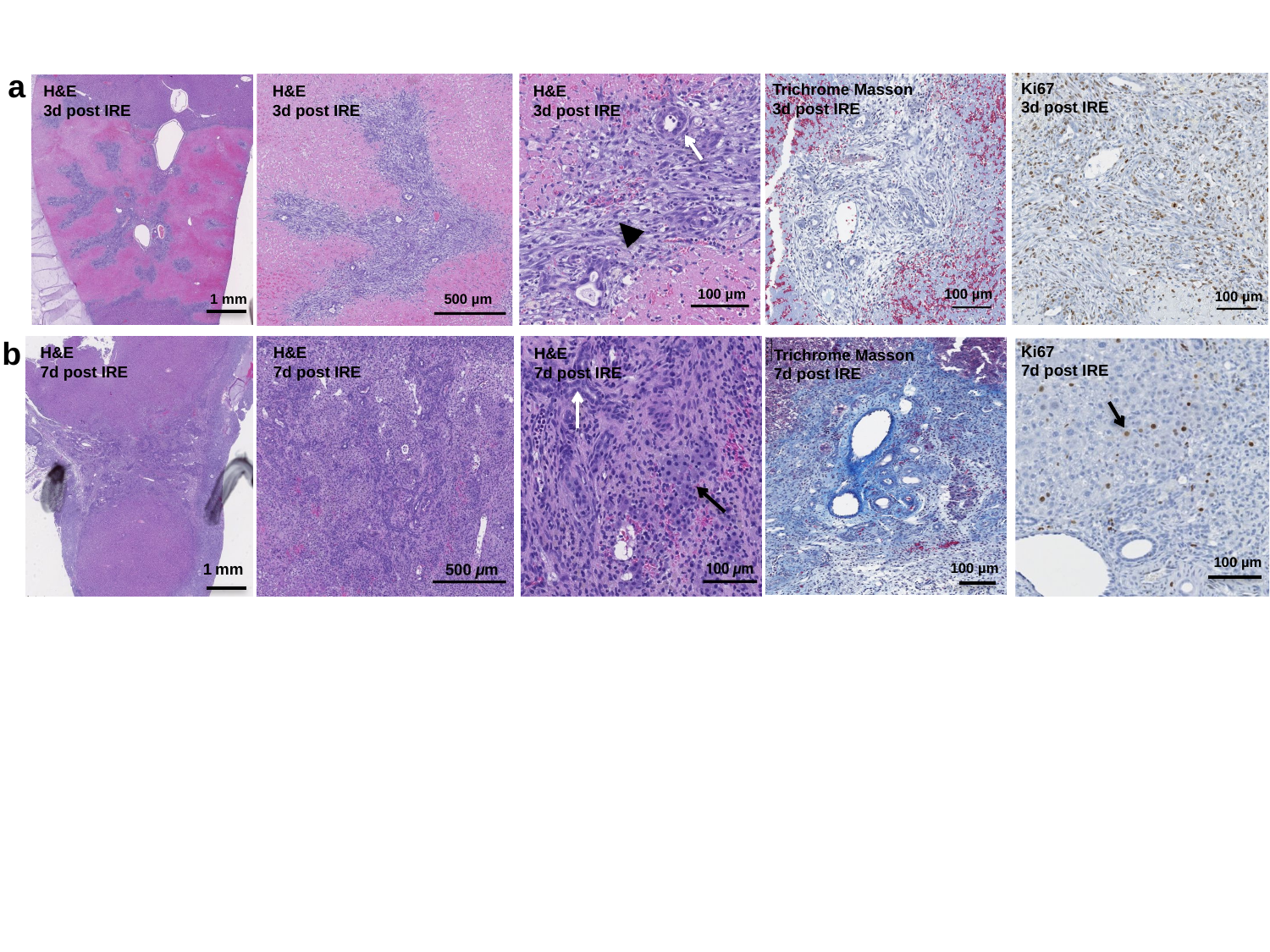

a
Ki67
3d post IRE
Trichrome Masson
3d post IRE
H&E
3d post IRE
H&E
3d post IRE
H&E
3d post IRE
100 µm
100 µm
100 µm
1 mm
500 µm
b
Ki67
7d post IRE
H&E
7d post IRE
H&E
7d post IRE
H&E
7d post IRE
Trichrome Masson
7d post IRE
100 µm
1 mm
100 µm

## Slide 3
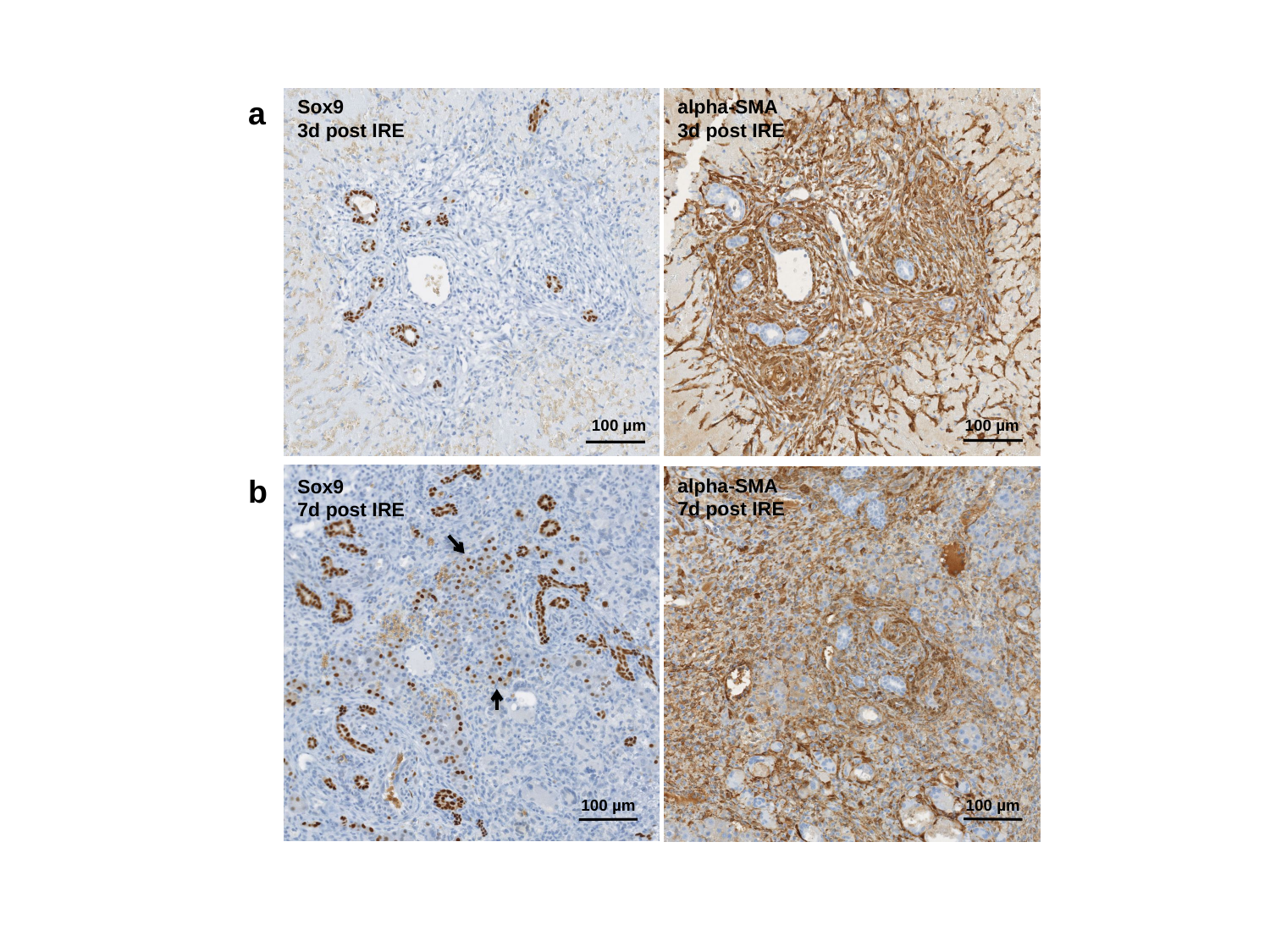

a
Sox9
3d post IRE
alpha-SMA
3d post IRE
100 µm
100 µm
b
alpha-SMA
7d post IRE
Sox9
7d post IRE
100 µm
100 µm

## Slide 4
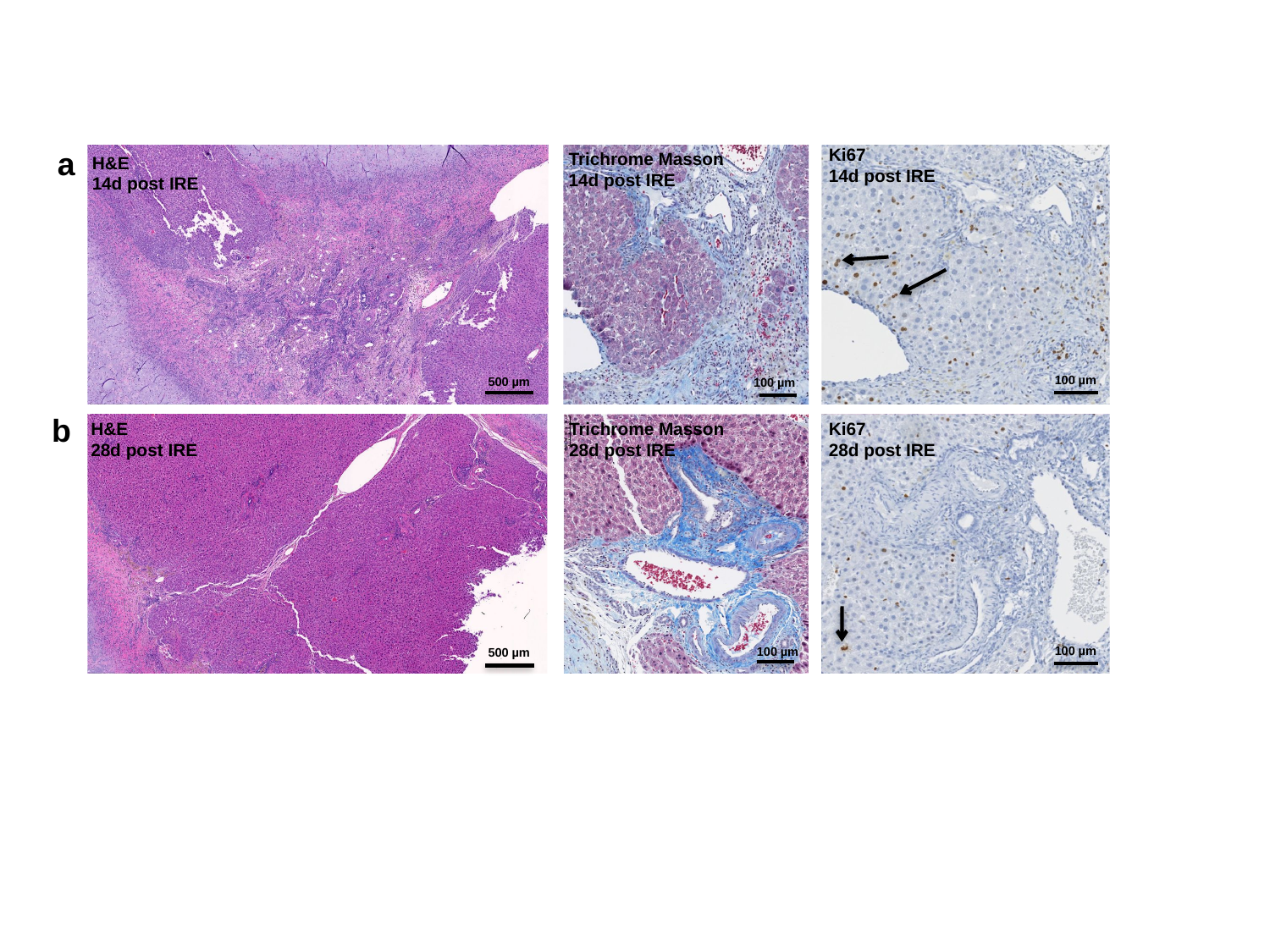

a
Ki67
14d post IRE
100 µm
Trichrome Masson
14d post IRE
H&E
14d post IRE
500 µm
100 µm
b
H&E
28d post IRE
Trichrome Masson
28d post IRE
Ki67
28d post IRE
100 µm
100 µm
500 µm

## Slide 5
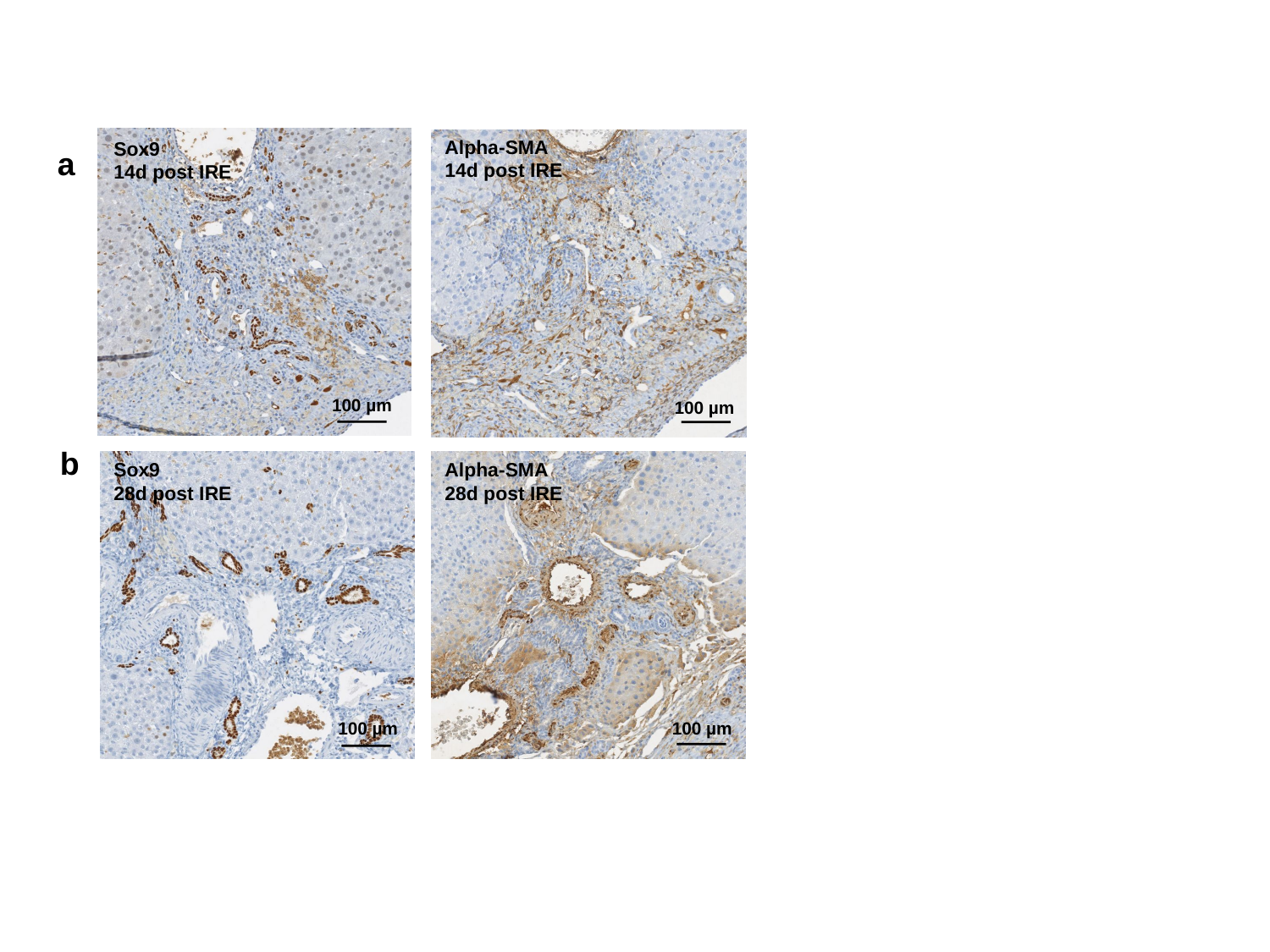

Alpha-SMA
14d post IRE
Sox9
14d post IRE
a
100 µm
100 µm
b
Sox9
28d post IRE
Alpha-SMA
28d post IRE
100 µm
100 µm

## Slide 6
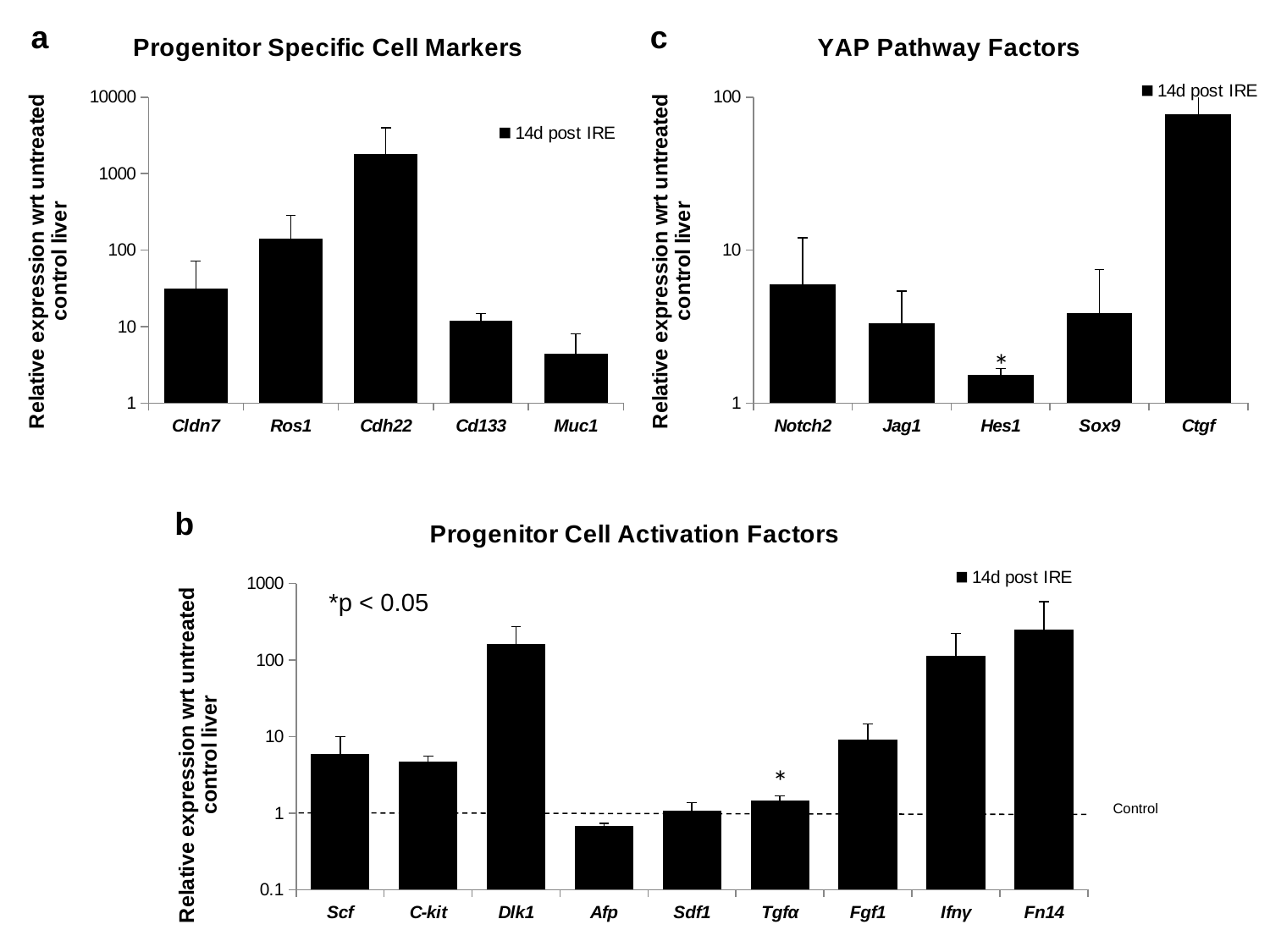

### Chart: Progenitor Specific Cell Markers
| Category | 14d post IRE |
|---|---|
| Cldn7 | 31.10446871141258 |
| Ros1 | 140.3730344980436 |
| Cdh22 | 1822.42929860006 |
| Cd133 | 11.80727866209899 |
| Muc1 | 4.479903964607762 |
### Chart: YAP Pathway Factors
| Category | 14d post IRE |
|---|---|
| Notch2 | 5.97942043405833 |
| Jag1 | 3.316391618467826 |
| Hes1 | 1.531597658903754 |
| Sox9 | 3.877381400400396 |
| Ctgf | 76.77500643816708 |c
a
*
*
### Chart: Progenitor Cell Activation Factors
| Category | 14d post IRE |
|---|---|
| Scf | 5.918948636108388 |
| C-kit | 4.6648612670896 |
| Dlk1 | 161.353358218068 |
| Afp | 0.681486083967775 |
| Sdf1 | 1.079703332015762 |
| Tgfα | 1.455173801173756 |
| Fgf1 | 9.241510793941883 |
| Ifnγ | 113.9428951948171 |
| Fn14 | 249.9093865538682 |b
*p < 0.05
*
Control

## Slide 7
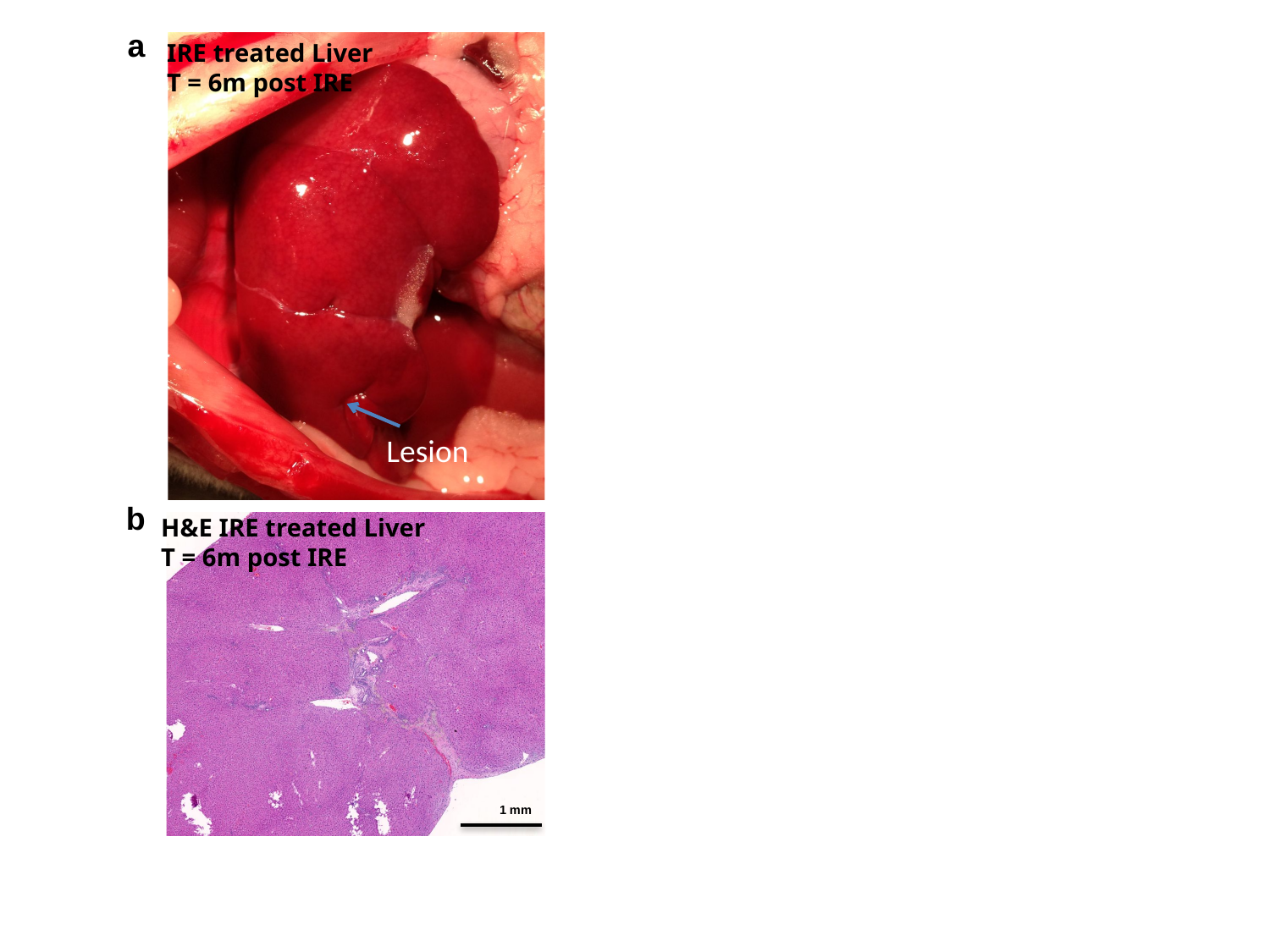

a
IRE treated Liver
T = 6m post IRE
Lesion 2
Lesion
b
H&E IRE treated Liver
T = 6m post IRE
1 mm

## Slide 8
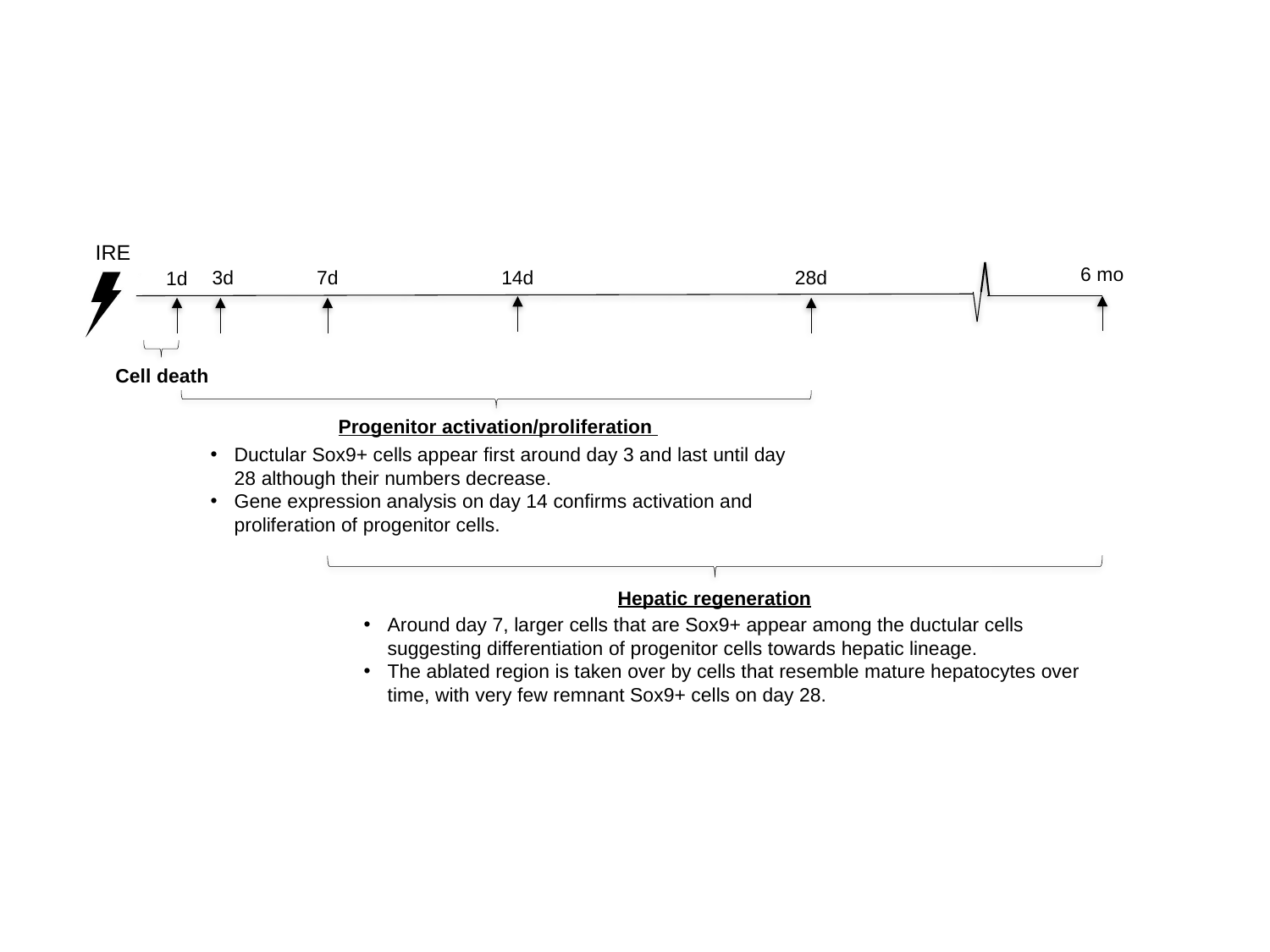

IRE
6 mo
7d
14d
28d
3d
1d
Cell death
Progenitor activation/proliferation
Ductular Sox9+ cells appear first around day 3 and last until day 28 although their numbers decrease.
Gene expression analysis on day 14 confirms activation and proliferation of progenitor cells.
Hepatic regeneration
Around day 7, larger cells that are Sox9+ appear among the ductular cells suggesting differentiation of progenitor cells towards hepatic lineage.
The ablated region is taken over by cells that resemble mature hepatocytes over time, with very few remnant Sox9+ cells on day 28.
